# Incorporating double copies of a chromatin insulator into lentiviral vectors results in less viral integrants

**DOI:** 10.1186/1472-6750-9-13

**Published:** 2009-02-24

**Authors:** Troels T Nielsen, Johan Jakobsson, Nina Rosenqvist, Cecilia Lundberg

**Affiliations:** 1 CNS Gene Therapy Unit, Wallenberg Neuroscience Center, Department of Experimental Medical Sciences, Lund University, BMC A11, S-221 84 Lund, Sweden; 2 Section of Neurogenetics, Department of Cellular and Molecular Medicine, Panum Instituttet, University of Copenhagen, Copenhagen, Denmark

## Abstract

**Background:**

Lentiviral vectors hold great promise as gene transfer vectors in gene therapeutic settings. However, problems related to the risk of insertional mutagenesis, transgene silencing and positional effects have stalled the use of such vectors in the clinic. Chromatin insulators are boundary elements that can prevent enhancer-promoter interactions, if placed between these elements, and protect transgene cassettes from silencing and positional effects. It has been suggested that insulators can improve the safety and performance of lentiviral vectors. Therefore insulators have been incorporated into lentiviral vectors in order to enhance their safety profile and improve transgene expression. Commonly such insulator vectors are produced at lower titers than control vectors thus limiting their potential use.

**Results:**

In this study we cloned in tandem copies of the chicken β-globin insulator (cHS4) on both sides of the transgene cassette in order to enhance the insulating effect. Our insulator vectors were produced at significantly lower titers compared to control vectors, and we show that this reduction in titer is due to a block during the transduction process that appears after reverse transcription but before integration of the viral DNA. This non-integrated viral DNA could be detected by PCR and, importantly, prevented efficient transduction of target cells.

**Conclusion:**

These results have importance for the future use of insulator sequences in lentiviral vectors and might limit the use of insulators in vectors for *in vivo *use. Therefore, a careful analysis of the optimal design must be performed before insulators are included into clinical lentiviral vectors.

## Background

Lentiviruses are complex retroviruses that are capable of infecting non-dividing cells[1,2]. As a consequence lentiviral vectors, such as those based on HIV-1, efficiently transduce a variety of cell types including embryonic stem cells, haematopoietic cells and neurons, and have been suggested as candidate vectors for both *in vivo *and *ex vivo *gene therapy applications [3-5]. A key step in the retroviral life-cycle is integration of the provirus into the host genome. This integrating nature of retroviruses has been exploited in the development of gene transfer vectors and has been deemed essential for certain gene therapy strategies, including *ex vivo *gene transfer[6,7]. However, adverse issues related to the integration of a provirus such as insertional mutagenesis, promoter interference and positional effects have been highlighted by induction of malignancy in mouse models[8] and by the development of leukaemia in five patients in two clinical gene therapy trials[9,10]. Consequently, obtaining adequate safety and efficiency of viral vectors is a focus of interest in vector development.

Insertion of chromatin insulator elements into the vector sequence has been proposed as a solution to problems related to the integration of proviruses[11]. An insulator is a *cis*-acting element characterized by one or both of the following criteria: it prevents enhancer-promoter interactions, if placed in between these elements, and protects transgenic expression cassettes from silencing and positional effects[11,12]. Hence, if these characteristics could be transferred to a lentiviral vector it would confer protection from influences of the integrated viral sequence to surrounding endogenous genes, and since it may prevent silencing of a proportion of the proviruses, it may also enhance vector functionality allowing a lower number of integrants to be used in order to achieve therapeutic efficacy. Taken together, such improvements of the vector properties would be likely to reduce the risk related to the proviral integration[13].

However, before insulators are included into clinical gene therapy vectors, a number of issues remain to be clarified. So far, no studies have clearly demonstrated an enhanced safety of insulator vectors, although it has recently been shown that inclusion of insulators in lentiviral vectors suppresses clonal dominance *in vitro *and partially protects against genotoxicity and promoter interference [14-17]. Furthermore, improved transgene expression from insulator containing retro- and lentiviral vectors has been reported predominantly in cell lines of erythroid origin and appears to be cell-type dependent [18-20]. Finally, the inclusion of insulator-sequences has been reported to reduce the titer of viral vector preparations, which complicates transduction of difficult target cells or direct *in vivo *applications[19,21,22].

In this study we addressed some of the issues of lentiviral vectors carrying chromatin insulators. For this we designed a set of insulator-lentiviral vectors that were tested in cells of different lineages. We found that lentiviral vectors are indeed capable of incorporating insulator sequences leading to efficient transgene expression in different cell types. However, a careful examination of their functionality suggests that the titer is compromised at a step that takes place after reverse transcription but before proviral integration. This finding has implications for the design of insulator-containing lentiviral vectors and highlights the importance of extensive studies of vector functionality following inclusion of *cis*-acting elements.

## Results

### Lentiviral vectors

**Figure 1:**
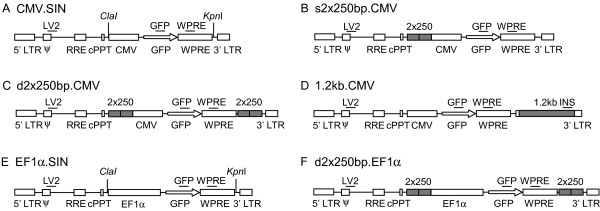
**Outline of lentiviral vectors**. Schematic drawing of vectors used in this study. Vectors A-D utilize the CMV-promoter whereas vectors E-F use the EF1α-promoter. (A, E) Control vectors for the group of CMV and EF1α vectors respectively. Cloning sites for the *cis*-elements are shown. (B) One tandem repeat (2 × 250) of the core element of chicken β-globin insulator (cHS4) was inserted (sense orientation) prior to the promoter. (C, F) Two copies of the tandem repeat were inserted into the vectors – one just upstream the promoter and one just downstream the expression cassette. (D) The entire cHS4-insulator (1.2 kb) was inserted in the 3' LTR in sense orientation. (A-F) Horizontal lines denote the location of primers (labelled LV2, GFP, WPRE and INS) used for quantitative real-time PCR (qPCR). Legend: LTR – Long Terminal Repeat, ψ – packaging signal, RRE – Rev Responsive Element, cPPT – Central Poly Purine Tract, CMV – Cytomegalo virus promoter, EF1α – Elongation Factor 1α promoter, GFP – Green Fluorescent Protein, WPRE – Woodchuck Posttranscriptional Regulatory Element, 2 × 250 – tandem repeat of the cHS4 core element and 1.2 kb – full length cHS4 insulator sequence.

We constructed second generation HIV-1 derived lentiviral vectors with the chicken β-globin insulator (cHS4) in various configurations (Figure 1). All vectors carried the central polypurine tract (cPPT) and the woodchuck hepatitis post-transcriptional regulatory element (WPRE), which have been shown to enhance the functionality of lentiviral vectors[23,24]. We used two promoters, the human cytomegalovirus promoter (CMV) and the human elongation factor 1α promoter (EF1α), in order to avoid misinterpretation of the results due to possible promoter-specific events. The first design of the insulator vectors had tandem copies of the 250 bp core element[25] placed just upstream of the expression cassette (s2 × 250 bp.CMV, figure 1B). The second design had a second doublet of the 250 bp core element placed just after the transcriptional cassette (d2 × 250 bp, figure 1C and 1F). We also used our previously reported vector, which carries the full length 1.2 kb insulator into the 3'LTR (1.2 kb.CMV, figure 1D)[21]. This design leads to a duplication of the insulator sequence into the 5' LTR during reverse transcription. As control vectors we used CMV.SIN and EF1α.SIN (Figure 1A and 1E).

### Titers of insulator-containing vectors are reduced

Previously, we and others have suggested that the functional titer is reduced when insulator sequences are incorporated into retroviral and lentiviral vectors[19,21,22]. Therefore, we decided to estimate vector titer in two independent ways. The functional titer was estimated by transducing 293T cells with limited dilutions of vector stocks. The proportion of GFP expressing cells was then estimated and transductions containing around 10% GFP expressing cells were used to calculate a functional titer. A relative DNA titer was measured by quantifying the level of viral DNA relative to endogenous genomic DNA by performing real-time PCR on DNA from transduced 293T cells.

The vector carrying a single copy of the 2 × 250 bp doublet (s2 × 250 bp.CMV) was produced at a similar functional titer as the control vectors (CMV.SIN and EF1α.SIN) (Table 1). However, the titer of our vector carrying the 1.2 kb (1.2 kb.CMV) insulator in the LTR was significantly reduced (P < 0.01) more than 6-fold (average: 4.9 × 10^7 ^TU/ml, 1.2 kb.CMV vector vs. 3.0 × 10^8 ^TU/ml, CMV.SIN) which is in agreement with what we have reported previously with a similar design lacking the cPPT[21]. In addition, the functional titer of the CMV vector carrying double copies of the 2 × 250 bp element (d2 × 250 bp.CMV) was significantly (P < 0.01) reduced almost 20-fold (average 1.6 × 10^7 ^TU/ml) compared to the control vector (CMV.SIN). The same was true for the d2 × 250 bp.EF1α vector where the functional titer was reduced almost 28-fold (1.4*10^7^TU/ml of d2 × 250 bp.EF1α vs. 3.9*10^8 ^of EF1α.SIN). Interestingly, such a dramatic reduction in the relative DNA titer could not be observed, where only the titer of d2 × 250 bp.EF1α was significantly reduced (4-fold, 9.0*10^7^TU/ml of d2 × 250 bp.EF1α vs. 3.9*10^8 ^of EF1α.SIN).

**Table 1:** Titer of viral batches

**Vector**	Functional titer *10^8 ^TU/ml	Relative DNA titer *10^8 ^U/ml
CMV.SIN	3.0 ± 0.4	N/A
s2 × 250.CMV	1.2 ± 0.5	1.6 ± 0.05
d2 × 250.CMV	0.16 ± 0.05 *	0.62 ± 0.05
1.2 kb.CMV	0.49 ± 0.08 *	1.2 ± 0.3
EF1α.SIN	3.9 ± 0.9	N/A
d2 × 250.EF1α	0.14 ± 0.02 *	0.9 ± 0.2 *

Functional and relative DNA titer of viral vectors (mean ± SEM). CMV.SIN and EF1α.SIN work as references for each group of vectors. By definition the relative DNA titers of the control vectors (CMV.SIN and EF1α.SIN) are identical to their respective functional titers. * denotes significant (P < 0.05) different titer compared to the vector's respective control.

In order to analyse if the discrepancy between functional titer and relative DNA titer of d2 × 250 bp and 1.2 kb.CMV vectors was due to loss of the transcriptional cassette by homologous recombination during the transduction process, we transduced 293T cells with the four vectors carrying the CMV promoter (Figure 1A–D). DNA was harvested at 28 days post-transduction, and real-time PCR was performed using four sets of primers (LV2-, GFP-, WPRE- and INS-primers, see figure 1 for primer location) to determine the abundance of each of the different sequences in relation to each other. The LV2-primer was used as reference. At this late time-point post transduction we expected that potential episome-like structures would be diluted out due to cell division and that only properly integrated viral sequences would contribute to the real-time PCR readings. We found an approximate 1:1-ratio between LV2-, GFP- and WPRE-sequences in all vectors but the one carrying two copies of the insulator doublet (d2 × 250 bp.CMV vector, Table 2). The latter showed a significant (P < 0.001) decrease in the abundance of the GFP-sequence when compared to the control vector (0.69 ± 0.02, d2 × 250 bp.CMV vs. 1.15 ± 0.05, CMV.SIN, relative units) along with an insignificant tendency that also the WPRE-sequence was underrepresented (0.88 ± 0.08, d2 × 250 bp vs. 1.25 ± 0.09, CMV.SIN, relative units). This suggests that only the d2 × 250 bp vector loses the transcriptional cassette during transduction but that this happens only in a minority of integrants. Such rearrangements are common in recombinant retroviral vectors with direct repeats[26,27], and our data suggest that the same might be true for some configurations of direct repeats in lentiviral vectors. However, as only a minority of the d2 × 250 bp.CMV integrants seems to have undergone rearrangements and since the other three vectors, including the 1.2 kb.CMV vector, were integrated in a proper configuration, rearrangements followed by loss of the transcriptional cassette do not seem to explain the discrepancy between the functional titer and the relative DNA titer. Finally, we found that the ratio between the QPCR readings of LV2 and INS was approximately 1:2 after transduction with the 1.2 kb.CMV vector compared to 1:1 in plasmid DNA (Table 2), showing that the full length 1.2 kb insulator is efficiently duplicated during reverse transcription.

**Table 2:** Integrity of proviral integrants

**Vector**	**LV2 primers (Reference)**	**Relative measurement** **GFP primers**	**Relative measurement** **WPRE primers**	**Relative measurement** **INS primers**
CMV.SIN	1	1.15 ± 0.05	1.25 ± 0.09	-
s2 × 250 bp.CMV	1	1.11 ± 0.02	1.39 ± 0.14	-
d2 × 250 bp.CMV	1	0.69 ± 0.02 *	0.88 ± 0.08	-
1.2 kb.CMV	1	1.20 ± 0.05	1.21 ± 0.29	2.23 ± 0.04 *
1.2 kb.CMV (Plasmid)	1	-	-	0.98 ± 0.03

Real-time PCR data showing the abundance of four sequences (see figure 1 for primer location) present in the viral vectors. 293T cells were transduced at MOI 1 (functional titer) and after 28 days cells were harvested and analysed. Abundance of sequences detected with primer pairs GFP, WPRE and INS was calculated using LV2 primers as reference. For CMV.SIN and s2 × 250 bp.CMV the ratio between LV2, GFP and WPRE was approximately 1:1:1 as expected. With respect to the d2 × 250 bp.CMV vector the ratio was roughly 1:0.7:0.9, which indicate that the GFP sequence is less abundant in cell cultures transduced with this vector (P < 0.001). This suggests that the vector undergoes some degree of rearrangement during production, reverse transcription or integration. In contrast, the 1.2 kb.CMV vector did not show any sign of rearrangements, as the ratio of LV2, GFP, WPRE and INS was approximately 1:1:1:2 when measuring on genomic DNA of transduced cells. In addition, the ratio between LV2 and INS was 1:1 when measuring on 1.2 kb.CMV plasmid DNA as expected. Significance is denoted by *.

In summary, the two different ways of titration and the analysis of possible vector rearrangement suggest that the vectors containing two separated copies of the insulator in the reversed transcribed form (d2 × 250 bp.EF1α, d2 × 250 bp.CMV and 1.2 kb.CMV) are fully functional and that the transgene expression from these vectors appears to be reduced compared to control vectors (EF1α.SIN, CMV.SIN and s2 × 250 bp.CMV).

### Transgene expression

To determine the influence of the insulator elements on transgene expression three different cell-types of different lineages were transduced with the lentiviral vectors at a multiplicity of infection (MOI) of 1 and 5 based on the relative DNA titer. The use of the relative DNA titer, which is not based on transgene expression capability during titration, allows comparison between different vector designs. For these experiments we used human 293T cells, which are derived from embryonic kidney, RN33B, which are immortalized rat neural precursor cells, and K562, which are human erythroid progenitor cells. The transduced cells were passaged for 7 days and then transgene expression (GFP) was determined by flow cytometry. The neural stem cell line, RN33B, was in a separate experiment also differentiated *in vitro *for 7 days.

**Figure 2:**
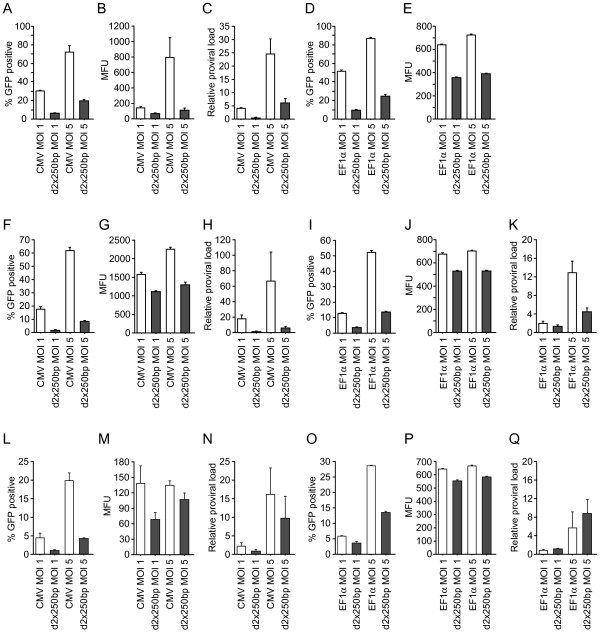
**Expression data of the d2 × 250 bp vectors**. Flow cytometric data and corresponding determination of proviral load for d2 × 250 vectors in three different cell types: RN33B (naïve) (A-E), 293T (F-K) and K562 (L-Q). Cells were transduced at MOI 1 and 5 (relative DNA titer) and analysed 7 days after transduction. The figure shows the percentage of GFP positive cells for each vector (A, D, F, I, L and O) along with the corresponding mean fluorescence (MFU) (B, E, G, J, M and P) and proviral load (the latter only for selected vectors) (C, H, K, N and Q). Error bars denote standard deviations.

When transducing the cell lines with the d2 × 250 bp vectors, we found less GPF-positive cells and lower mean fluorescence (mean fluorescence units – MFU) independent of cell type and promoter choice when comparing with control vectors (Figure 2A–Q), which suggests that the cHS4 core element fails to increase transgene expression. In addition, the insulator was unable to prevent transgene silencing during differentiation of RN33B cells (results not shown). This is in agreement with recent results showing that the 250 bp core element of the cHS4-insulator is insufficient for insulating properties in a retroviral and lentiviral context[15,28]. Surprisingly, however, QPCR analyses of the proviral load in transduced cell cultures revealed significantly lower levels of viral DNA in cultures transduced with the d2 × 250 bp vectors compared to control vectors (Figure 2, C, H, K and 2N), despite the fact that we had normalized the various transductions using the relative DNA titer. This difference in viral DNA accounted for most of the difference in transgene expression. This is surprising since we have previously used the relative DNA titer for titer determination of vectors with another commonly used *cis*-element, the scaffold attachment region (SAR)[19,29], and subsequently found complete agreement between the proviral loads of cell cultures transduced with the vector containing this *cis*-element and the control vector (supplementary information (Additional file 1 and 2: Supplementary information.pdf and Supplementary tables.pdf)). In addition, these data confirm that the negative effects on vector functionality observed when inserting the 2 × 250 bp doublet after the WPRE are not caused by the size increment of the vector, since the insertion of the SAR element, which is similar in size compared to the 2 × 250 bp doublet, in this position does not influence the functional titer, the relative DNA titer, transgene expression levels or proviral loads of transduced cultures. Thus the reduced transgene expression from the d2 × 250 bp vectors probably reflects impaired vector functionality with regard to cell entry, integration etc, and this might explain the observed discrepancies between the functional titer and the relative DNA titer.

### Double copies of insulators result in less proviral integrants

One explanation of the overestimation of titer when using relative DNA titration of the d2 × 250 bp and 1.2 kb.CMV vectors could be that these configurations of insulator sequences have a deleterious effect on vector functionality at a stage after reverse transcription (i.e. viral integration). Using real-time PCR to quantify viral DNA detects not only viral DNA that is properly integrated but also viral DNA that remains non-integrated. If such non-integrated viral DNA was abundant in cell cultures transduced with the d2 × 250 bp and the 1.2 kb.CMV vectors, titer determination using real-time PCR 3 days after transduction would overestimate the titers, compared to titer determination conducted 7 days post-transduction, where non-integrated viral DNA will be more diluted due to ongoing cell division. In this study titer determination was performed 3 days post transduction where all viral integration has normally occurred[30], whereas viral DNA levels in the expression analyses were measured 7 days post transduction. A similar overestimation of the functional titer is unlikely to happen if the non-integrated viral DNA is present in a form not suitable for transgene expression, since only properly integrated and transgene expressing vector genomes will contribute to this titer determination. These circumstances could explain the inconsistent relationship between the relative and the functional titer and the proviral loads measured in the expression analyses of the d2 × 250 bp and the 1.2 kb.CMV vectors.

**Figure 3:**
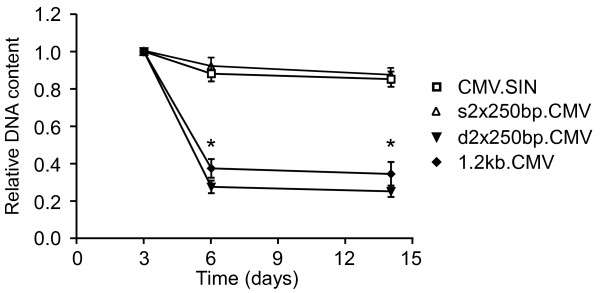
**Persistence of viral DNA in transduced cells during a 14 day time period**. 293T cells were transduced at MOI 1 (relative DNA titer) with the four vectors utilizing the CMV-promoter. After 3, 6, 14 and 27 days cells were harvested and the proviral load determined by qPCR using primers LV2 and ALB. The vectors containing two separated copies of the insulator (d2 × 250 bp.CMV and 1.2 kb.CMV) elicit a significant (*, p < 0.05) drop in proviral load within the first 6 days after transduction compared to control vectors, CMV.SIN and s2 × 250 bp.CMV. The experiment was continued for 27 days with no change in proviral load compared to the 14 days time point. Error bars denote standard error of the mean.

To test this hypothesis we transduced 293T cells at MOI 1 (based on functional titer) with the four CMV vectors. Cells were passaged and part of each cell culture was harvested at days 3, 6, 14 and 27. Real-time PCR (using primers LV2 and ALB) on DNA isolated from these cells revealed that the proviral load was significantly (P < 0.001) decreased in cells transduced with the d2 × 250 bp vector and the 1.2 kb vector during day 3–6 (on day six the proviral loads of the d2 × 250 bp vector and the 1.2 kb vectors were 28% ± 10% and 37% ± 16%, respectively, of their initial proviral loads measured on day three) (Figure 3). After the initial drop the proviral load stayed a constant low level for at least 27 days (only 3, 6 and 14 days time points are shown in the figure). In vectors containing none or only one insulator-doublet in the reverse transcribed form (CMV.SIN and s2 × 250 bp.CMV) no such drop was observed (Figure 3). These results support the hypothesis that lentiviral vectors carrying two separated copies of the cHS4 insulator in the reverse transcribed form are capable of efficient cell-entry and subsequent reverse transcription (since high levels of viral DNA can be detected 3 days post transduction), but that the transduction process is impaired at some step after reverse transcription but before integration (since the abundance of viral DNA declines during day 3–6 post transduction). Notably, the insulator sequence itself is not deleterious to the vector since we could not detect any decrease in viral load of the s2 × 250 bp.CMV vector from day 3 to 6 (Figure 3). The same was true for our vector containing the SAR element confirming that loss of viral DNA from day 3–6 was not attributable to the size increment of the d2 × 250 bp vector (supplementary figure 3 (Additional file 3: Supplementary figures.pdf)). It is possible that two identical insulator sequences separated by an appropriate spacing as in the d2 × 250 bp.CMV vector and the reversed transcribed form of the 1.2 kb.CMV vector induce the formation of secondary structure incompatible with proper transduction and transgene expression.

### Non-integrating vectors prevent efficient transduction

Our results with the d2 × 250 bp vectors suggest that the low titer is due to several independent defects. As shown above, a large proportion of the vectors are unable to integrate and express the transgene. Furthermore, a minor proportion of the vectors that integrate will have lost the transgene cassette due to vector rearrangements. However, it is worth pointing out that in contrast to oncoretroviral vectors with a similar design functional particles are produced[22].

**Figure 4:**
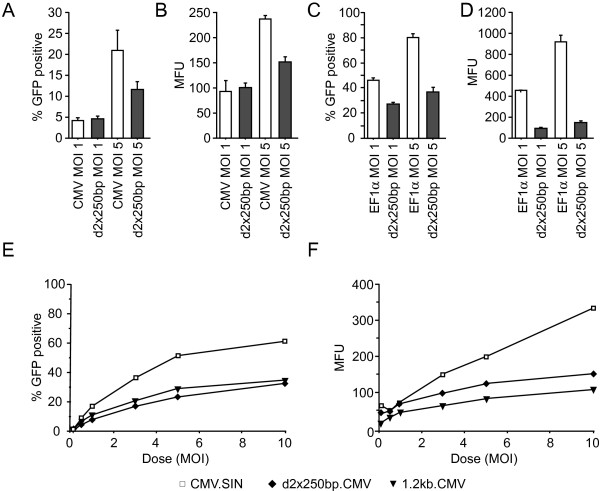
**Expression data of the d2 × 250 bp vectors**. (A-D) Flow cytometric data for the transduction of naïve RN33B cells with d2 × 250 bp vectors and controls at MOI 1 and 5 (functional titer). Cells were analysed 7 days after transduction. The percentage of GFP-positive cells is shown (A and C) along with the mean fluorescence (MFU) (B and D). Error bars denote standard deviations. E-F: Dose-response curves showing CMV.SIN, d2 × 250 bp.CMV and 1.2 kb.CMV vector performance in naïve RN33B cells. The percentage of GFP positive cells (E) and mean fluorescence is shown (F).

To test the transduction potential of these remaining functional vectors, we transduced RN33B cells at an MOI of 1 and 5, this time based on the functional titers. At 7 days after transduction cells were harvested and flow cytometry performed. For the CMV vectors at a MOI of 1, the d2 × 250 bp vector seemed to perform as well as the control vector (Figure 4A and 4B). However, at high MOI, the d2 × 250 bp vector expressed GFP at a much lower level than the control vector (Figure 4A and 4B). When using EF1α vectors the performance of the d2 × 250 bp vector was even worse compared to the control vector (Figure 4C and 4D).

We then transduced the neural stem cell line RN33B with increasing doses of lentiviral vectors (CMV.SIN, d2 × 250 bp.CMV and 1.2 kb.CMV) based on functional titer. At low MOIs the difference in expression between control vectors and insulator vectors was relatively small both with regard to the number of GFP positive cells and the fluorescence intensity (Figure 4E–F). However, at higher MOIs the dose-response curve of the insulator vectors reached plateau at a lower number of transduced cells and lower fluorescence intensity (Figure 4E–F). As to why this reduced expression is due to a block in the transduction pathway remains uncertain, but nonetheless efficient transduction and high levels of transgene expression from these vectors seem difficult to achieve.

## Discussion

The original intention of this study was to analyse the effects of a chromatin insulator on transgene expression when inserted into lentiviral vectors. The idea was to use a set-up that was independent of any type of selection and that would allow us to study potential insulator effects in cells with multiple integrants. Up to date most studies using insulator sequences in retroviral or lentiviral settings have relied on drug selection of transgene expressing cells[19,28,31]. Furthermore, most studies have been designed so that only cells with single copy integrants have been analysed[18,19,32,33]. Such settings have little in common with clinical gene therapy protocols. In order to perform a non-selection based experiment we decided to titer the vectors using real-time PCR on DNA from transduced cells. This protocol has the advantage that it does not rely on transgene expression in order to quantify the titer of the vector preparation and allows a non-biased quantification of integrating particles. When using standard lentiviral vectors without or with only one insulator element we found highly reproducible results with this non-selection based strategy. However, when we tried to apply our non-biased strategy onto vectors containing two separated copies of the cHS4 insulator in the reversed transcribed form, we found that the DNA titration led to overestimation of the titer, supposedly because of the formation of secondary structure that is incompatible with efficient transduction. This block appears to take place in a step during transduction that is after reverse transcription but before integration. How this block of integration is established is unknown but it is possible that potential secondary structures, caused by the double copies of the insulator, impede proper formation and function of the pre-integration complex (PIC) e.g. by preventing correct alignment of the integrase to the integrase binding sites or by hampering proper trafficking of the viral genome to the nucleus after infection. However, several recent reports demonstrate efficient transgene expression from non-integrating lentiviral vectors present as classical 1-LTR or 2-LTR circles[34,35]. These studies describe long-term expression in post-mitotic cells at levels comparable to integrating vectors. Since we show reduced expression from double copy insulator vectors, the non-integrated vector copies that we demonstrate must exist in a different form incompatible with transgene expression.

These results have important consequences for the use of insulator sequences in lentiviral vectors. It has been suggested that insulators need to be present in double or more copies in order to be fully functional[36,37]. This has led to the suggestion that insulators form a loop-structure that is resistant to influences from surrounding chromatin. However, lentiviral and retroviral vectors carrying such double copies have been reported by several groups to be produced in low titer[15,19-22]. As we demonstrate in this study that phenomenon is due to a block during the infectious process, and, importantly, this seems to prevent efficient transduction. When target cells were transduced with increasing doses of vector, the resulting dose-response curve reached a plateau at a lower number of transduced cells and with a markedly reduced mean fluorescence compared to control vectors. This suggests that efficient transduction of difficult target cells such as primary cells will be compromised with this kind of insulator vectors, which will hamper their use in both *ex vivo *and *in vivo *gene therapy settings.

We and others have seen only a modest effect on transgene expression when using insulators in lentiviral vectors[14,19,21]. On the contrary, there appears to be strong positive effects when using more simple retroviral vectors such as those based on MLV[18,28,33], and it is likely that the effects of an insulator will be dependent on the context, i.e. MLV contra lentiviral vectors. MLV-based and lentiviral vectors have different integration patterns. While MLV vectors preferentially integrate near the promoter region, the HIV-based vectors appear to integrate into the entire intragenic region[38,39]. It is not certain how these differences in integration sites affect transgene expression or the appearance of insertional mutagenesis. However, recent experiments in a tumorigenic mouse model show that self-inactivating lentiviral vectors have low genotoxicity compared to LTR-driven MLV vectors suggesting an enhanced safety profile of such lentiviral vectors[40]. Furthermore, lentiviral vectors appear to escape transgenic silencing more efficiently than MLV vectors. An example of this is the establishment of transgenic animals using lentiviral vectors[41], although several examples of lentiviral silencing in the generation of transgenic animals have also been reported[42].

Other *cis*-elements that have successfully been used to enhance transgene expression from lentiviral vectors have been identified, e.g. scaffold or matrix attachment regions (SAR, MAR or S/MARs). These are A+T rich elements that have been suggested to be involved in the anchoring of the chromatin to the nuclear scaffold resulting in the formation of chromatin loops thereby avoiding gene silencing[43,44], and elements that have shown to prevent transgene silencing in a lentiviral context have been derived from the human interferon-β gene, the human immunoglobulin μ heavy-chain locus, the immunoglobulin κ, etc[19,45,46]. Furthermore, the use of S/MAR in combination with insulators have been shown to be highly effective in enhancing transgene expression from lentiviral vectors[19,47]. However, the effect of the S/MAR elements appears, in most cases, to be tissue specific (for review see[48]), which limits their general application, and identification of S/MAR elements active in neural cells will be important for gene therapeutic applications in the brain. Improving lentiviral vector characteristics with insulators or other *cis*-elements is therefore still an area of interest.

## Conclusion

In conclusion our results demonstrate that insulator sequences can be incorporated into lentiviral vectors either in the LTR or flanking the transgene cassette. However, when double copies are used these vectors have a reduced functionality due to a block during the infectious process, and consequently such vectors transduce target cells poorly. Therefore, a careful analysis of the optimal design must be performed before insulators are included into clinical lentiviral vectors. Furthermore, it still remains to be proven that insulator sequences actually improve the safety profile of lentiviral vectors.

## Methods

### Transfer plasmids

The 250 bp core element of the cHS4 insulator was amplified from plasmid pJC5-4 (kindly provided by G. Felsenfeld, Laboratory of Molecular Biology, National Institute of Diabetes and Digestive Kidney Diseases, National Institute of Health, Bethesda) by four PCR-reactions using the following PCR-reaction: 3 min at 95°C followed by 30 cycles of 30 sec at 95°C, 45 sec at 55°C and 90 sec at 72°C. Primers were designed according to Chung et al.[25] and contained additional restriction sites (ClaI, KpnI and MluI) making it possible to join two 250 bp core elements into a 500 bp doublet where each core element was situated in the same direction. Primers were as follows: Kpn up: 5'-GGT ACC GGA GCT CAC GGG GAC AGC-3' Kpn down: 5'-GGT ACC CCT AAA GCT TTT TCC CCG TA-3' Cla up: 5'-ATC GAT GGA GCT CAC GGG GAC AGC-3' Cla down: 5'-ATC GAT CCT AAA GCT TTT TCC CCG TA-3' Mlu up: 5'-ACG CGT GGA GCT CAC GGG GAC AGC-3' and Mlu down: 5'-ACG CGT CCT AAA GCT TTT TCC CCG TA-3'. Two doublets were generated: One was flanked by KpnI-sites whereas the other was flanked by ClaI-sites. These two fragments were then cloned in the sense orientation into the ClaI and the KpnI-sites of pHR-CMV.GFP.W (CMV.SIN) (kind gift from D. Trono, Lausanne, Switzerland) and pHR-EF1α.GFP.W (EF1α.SIN) (kindly provided by N.B. Woods, Lund, Sweden) using standard cloning procedures to generate pHR-2 × 250 bp.CMV.GFP.W.2 × 250 (d2 × 250 bp.CMV) and pHR-2 × 250 bp.EF1α.GFP.W.2 × 250 (d2 × 250 bp.EF1α). In addition a vector containing only one insulator doublet was constructed (pHR-2 × 250.CMV.GFP.W, s2 × 250 bp.CMV). The vector pHR-CMV.GFP.W.1.2 kb (1.2 kb.CMV) has been described elsewhere and was only modified by the addition of the cPPT fragment[21].

Lentiviral vectors were produced as described previously[4]. Briefly, transfer plasmids were co-transfected with pMD.G and pBRΔ8.91 into 293T-cells, supernatants were collected on days 3 and 4 and concentrated by ultracentrifugation. Viral vector preparations were not treated with DNAse during the production step, since this has been shown not to influence the functional titer or the relative DNA titers[49] (and unpublished observations). We also excluded different levels of contaminating plasmid DNA in the various vector preparations by performing real-time PCR directly on concentrated viral vector stocks and found no difference between the plasmid content of control vectors compared to insulator vectors (P = 0.52, data not shown). All results were reproduced with several independently produced viral batches.

### Determination of viral titer

Functional titers of vector preparation were assessed by transduction of 100,000 cells (293T) with serial dilutions of vector stocks. A dilution resulting in less than 10% GFP-positive cells was used for determining the functional titer of each vector. Each vector was titrated against the control vectors CMV.SIN or EF1α.SIN.

Relative DNA titers were assessed by transduction of 50,000 cells (293T) with serial dilutions of vector stocks. After 72 h cells were collected and lysed and subjected to Taqman PCR[21] using the following primers LV2-F: 5'-ACT TGA AAG CGA AAG GGA AAC-3', LV2-R: 5'-CAC CCA TCT CTC TCC TTC TAG CC-3' LV2-Probe: 5'-Fam-AGC TCT CTC GAC GCA GGA CTC GGC-Tamra-3'. All samples were analysed in triplicates. Using the comparative C_T_-method (userbulletin#2, ) the relative DNA-titers were calculated in relation to the control vectors, CMV.SIN and EF1α.SIN. By definition the relative DNA-titer of CMV.SIN and EF1α.SIN equalled their determined functional titer.

### Cell culturing, transduction, flow cytometric analyses and determination of proviral load

293T cells and K562 were cultured at 37°C in humidified atmosphere and 5% CO_2 _in DMEM and RPMI 1640 respectively. Media was supplemented with L-glutamine and 10% foetal bovine serum. RN33B and HiB5 cells were cultured and differentiated as described elsewhere[50].

Cells were transduced simply by applying the appropriate amount of viral stock solution directly to the media of cell cultures.

Flow cytometric analyses were performed as described by Jakobsson et al[21]. Proviral loads of analysed cell cultures were determined by real-time PCR. Briefly, cells were harvested and lysed in 18 μl 50 mM Tris-HCl, pH 8.2 (Amresco), 100 mM NaCl (Merck), 5 mM EDTA (Chemicon) and 0.5% SDS (Chemicon). After addition of 2 μl proteinase K (10 mg/ml, Gibco BRL) samples were incubated at 55°C for 30 minutes. 200 μl of water was added and samples were boiled for 10 minutes. Proviral load was assessed by real-time PCR using the LV2 primer (above) in reference to endogenous genes (the IL-2 gene in rat cells and the albumin, ALB, gene in human cells). Sequences of the IL-2 and albumin primers are: IL-2 Forward, 5'-GCC TTG TGT GTT ATA AGT AGG AGG C-3', IL-2 Reverse, 5'-AGT GCC AAT TCG ATG ATG AGC-3', IL-2 Probe, 5'-Fam-TCT CCT CAG AAA TTC CAC CAC AGT TGC TG-Tamra-3', ALB Forward, 5'-TGA AAC ATA CGT TCC CAA AGA GTT T-3', ALB Reverse, 5'-CTC TCC TTC TCA GAA AGT GTG CAT AT-3' and ALB Probe, 5'-Fam-TGC TGA AAC ATT CAC CTT CCA TGC AGA-Tamra-3'.

### Testing vector integrity

Quantitative real-time PCR was performed on cell lysates 28 days after infection as described above. Primers targeting a viral sequence (LV2, see above), GFP, WPRE and 1.2 kb insulator (INS) were used. Sequences of primers and probes were: GFP Forward, 5'-ACT ACA ACA GCC ACA ACG TCT ATA TCA-3, GFP Reverse, 5'-GGC GGA TCT TGA AGT TCA CC-3', GFP Probe, 5'-Fam-CGA CCA GCA GAA GAA CGG CAT CA-Tamra-3', WPRE Forward, 5'-CCG TTG TCA GGC AAC GTG-3', WPRE Reverse, 5'-AGC TGA CAG GTG GTG GCA AT-3', WPRE Probe, 5'-Fam-TGC TGA CGC AAC CCC CAC TGG T-Tamra-3', INS Forward, 5'-ACC GCT CTT TGG AGA AGG TAA A-3', INS Reverse, 5'-ATG AGA GAT AAT GGC CTT ACG TTG T-3', and INS Probe, 5'-Fam-CTT GCT AAA TCC AGC CCG ACC CTC C-Tamra-3'. Standard curves for each primer pair were constructed and the abundance of each sequence was calculated using the comparative Ct method, taking into account the efficiency of each primer set according to Pfaffl (2001)[51]. All primer pairs used for real-time PCR were validated and showed efficiencies above 90%.

### Statistics

All experiments were performed in triplicate and repeated in two independent experimental rounds and with independently produced viral batches. Flow cytometry and qPCR analyses were subjected to analysis of variance (ANOVA) using SAS software followed by Tukey's HSD when appropriate.

## Authors' contributions

TTN and JJ carried out the experiments, analysed data, and were primarily responsible for the preparation of the manuscript. NR carried out some of the cell culture work and data analyses. CL led the project, initiated the project and helped in writing the manuscript. All authors read and approved the final manuscript.

## Supplementary Material

Additional file 1 **Supplementary information. Information on cloning and testing of a lentiviral vector containing the scaffold attachment region (SAR) of the human interferon-β gene.** Click here for file

Additional file 2 **Supplementary tables.** Tables showing results from the titration of the SAR-containing vectors and results from the analyses of the proviral loads in cell cultures transduced with SAR-containing vectors. Click here for file

Additional file 3 **Supplementary figures.** Figures showing the SAR-containing vector and results from testing the vector. Click here for file
