# Supplementary material for: Incorporating double copies of a chromatin insulator into lentiviral vectors results in less viral integrants
Source: BMC Biotechnol. 2009 Feb 24;9:13. doi: 10.1186/1472-6750-9-13 (PMC2651870; doi:10.1186/1472-6750-9-13)
Supplement: Additional file 1 — Supplementary information. Information on cloning and testing of a lentiviral vector containing the scaffold attachment region (SAR) of the human interferon-β gene. [file 1472-6750-9-13-S1.pdf]

Supplementary information

Cloning of the scaffold attachment region into the lentiviral vector

The sequence for the SAR-element was amplified by PCR. Primers (containing additional restriction sites: KpnI) were as follows: SAR up: 5'-GCTGTTGGTACCTTGTCAACCTC-3' and SAR down: 5'-TGGGGTAAAGTACGGTACCCATCA-3'. Plasmid pTZ-E20 (kindly provided by J. Bode, Gesellshaft für Biotechnologishe Forshung, Braunsweig-Stöckheim, Germany) was used as template in the following PCR-reaction: 3 min at 95ºC followed by 30 cycles of 45 sec at 95ºC, 30 sec at 55ºC and 90 sec at 72ºC. After subcloning into a TA vector (Invitrogen) the fragment of 800bp was sequenced and released by KpnI-digestion and inserted into the KpnI-site of the lentiviral transfer vector pHR-CMV.GFP.W (CMV.SIN) (kind gift from D. Trono, Lausanne, Switzerland) in the anti-sense orientation to generate pHR-CMV.GFP.W.SAR (CMV.SAR). The vector can be seen in supplementary figure 1 (Additional file 2: Supplementary figures.pdf).

Vector testing

The titer of the CMV.SAR vector was determined by the two different approaches already described (titers can be seen in supplementary table 1 (Additional file 3: Supplementary tables.pdf)). Subsequently, cell cultures (293T, RN33B and K562 cells) were transduced at MOI 1 and 5 (relative DNA titer) with either the control vector (CMV.SIN) or the SAR vector (CMV.SAR). 7 days after transduction cells were harvested and flow cytometry was performed. Subsequently, the DNA of the harvested cell was subjected to QPCR using LV2, ALB and IL-2 primers to determine the proviral load in the cell cultures relative to each other. Results showed that the SAR vector was produced with a functional titer similar to the control vector and that the relative DNA titer and the functional titer corresponded well with each other (Supplementary table 1 (Additional file 3: Supplementary tables.pdf)). Furthermore, in general the SAR vector performed

as well as the control vector with respect to GFP expression, and the cell cultures transduced with the control vector and the CMV.SAR vector showed equal proviral loads when transduced at the same MOI (Supplementary figure 2 (Additional file 2: Supplementary figures.pdf)) and supplementary table 2 (Additional file 3: Supplementary tables.pdf)). This is in sharp contrast to the results obtained from the d2x250bp vectors that showed significantly reduced proviral loads after 7 days in culture compared to the control vector, although transduced at the same MOI (relative DNA titer) (Supplementary table 2 (Additional file 3: Supplementary tables.pdf)).

To test the persistence of viral DNA of the CMV.SAR vector 293T cells were transduced at MOI 1 (relative DNA titer) with CMV.SAR, d2x250pb.CMV and 1.2kb.CMV. Cells were harvested after 3, 6, 14 and 27 days and DNA was subjected to real-time PCR using primers LV2 and ALB to determine the proviral loads of each cell culture. As depicted in supplementary figure 3 (Additional file 2: Supplementary figures.pdf) the two vectors containing two separated copies of the insulator (d2x250pb.CMV and 1.2kb.CMV) showed a significant drop in proviral loads from day 3 to day 6, whereas the CMV.SAR vector stayed at initial levels throughout the experiment.
