# Supplementary material for: Incorporating double copies of a chromatin insulator into lentiviral vectors results in less viral integrants
Source: BMC Biotechnol. 2009 Feb 24;9:13. doi: 10.1186/1472-6750-9-13 (PMC2651870; doi:10.1186/1472-6750-9-13)
Supplement: Additional file 2 — Supplementary tables. Tables showing results from the titration of the SAR-containing vectors and results from the analyses of the proviral loads in cell cultures transduced with SAR-containing vectors. [file 1472-6750-9-13-S2.pdf]

Supplementary table 1 - Titer of viral batches

Vector	Functional titer *10^8 TU/ml	Relative DNA titer *10^8 U/ml
CMV.SIN	3.0±0.4	N/A
CMV.SAR	4.0±0.3	2.6±1.4

Titers of viral batches of the control vector CMV.SIN and the corresponding batches of the vector containing the SAR-element. By definition the relative DNA titer of the control vector is identical to the functional titer. The titers of the two types of vectors are not significantly different (P>0.69).

Supplementary table 2 - Relative proviral loads in transduced cell cultures

Vector	MOI 1	MOI 5
CMV.SIN	1.2±0.1	5.9±0.4
CMV.SAR	1.5±0.2	6.4±1.1
CMV.SIN	1.0±0.1	4.5±0.9
d2x250bp.CMV	0.19±0.05 *	1.0±0.3*

Relative proviral load in cell cultures measured by QPCR 7 days after transduction with the CMV.SIN vector, the CMV.SAR vector or the d2x250bp.CMV vector at MOI 1 or MOI 5 (based on the relative DNA titer). As expected the CMV.SAR vector shows a proviral load that equals the proviral load of the control vector (CMV.SIN) at both MOI 1 and MOI 5 (P>0.16). As opposed to this, the d2x250bp.CMV vector shows a reduction of proviral load at both MOI 1 and MOI 5 of approximately 80% compared to the control vector CMV.SIN. * denotes significance, P<0.01.
