# Supplementary material for: Incorporating double copies of a chromatin insulator into lentiviral vectors results in less viral integrants
Source: BMC Biotechnol. 2009 Feb 24;9:13. doi: 10.1186/1472-6750-9-13 (PMC2651870; doi:10.1186/1472-6750-9-13)
Supplement: Additional file 3 — Supplementary figures. Figures showing the SAR-containing vector and results from testing the vector. [file 1472-6750-9-13-S3.pdf]

Supplementary figure 1 - **Outline of the CMV.SAR vector**

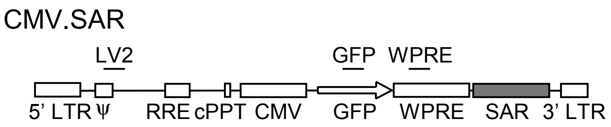

Schematic representation of the SAR-containing vector. The SAR element is situated in anti-sense direction.

Supplementary figure 2 - Expression data of the CMV.SAR vector

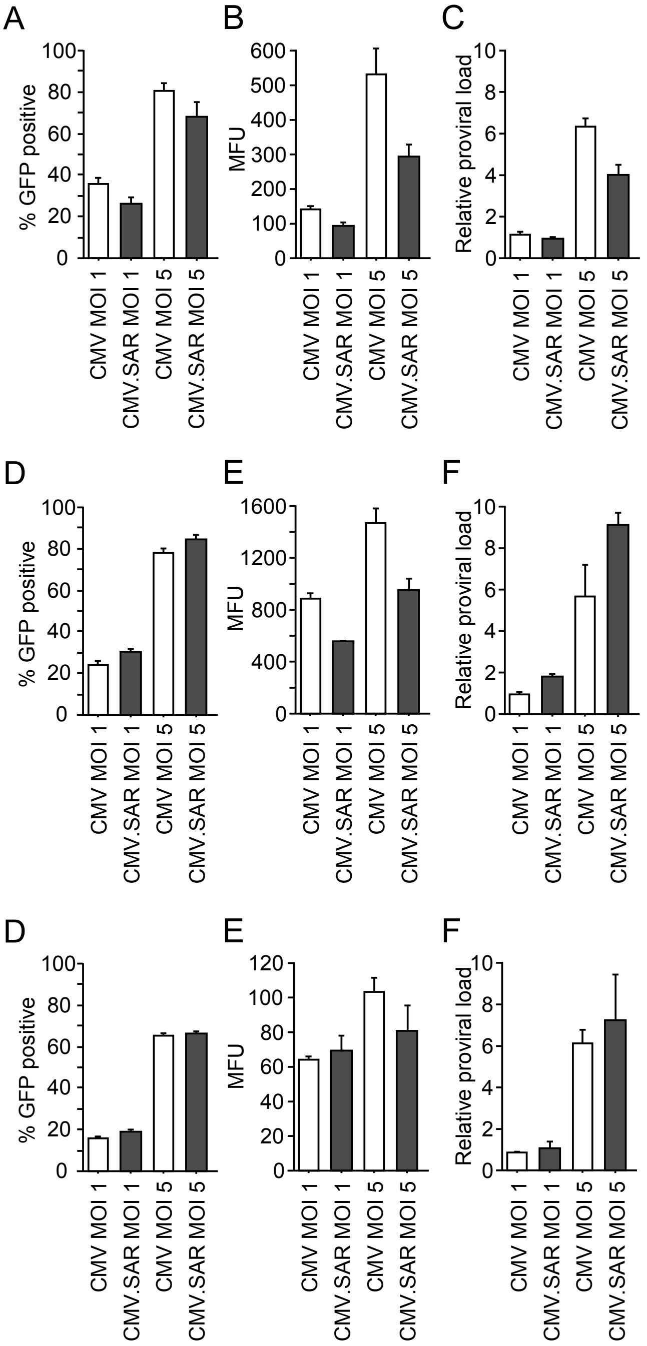

Flow cytometric data and corresponding determination of proviral load for the CMV.SAR vector in three different cell types: RN33B (naïve) (A-C), 293T (D-F) and K562 (G-I). Cells were transduced at MOI 1 and 5 (relative DNA titer) and analysed 7 days after transduction. The figure shows the percentage of GFP positive cells for each vector (A, D and G) along with the corresponding mean fluorescence (MFU) (B, E and H) and proviral load (C, F and I). Error bars denote standard deviations.

Supplementary figure 3 - Persistence of viral DNA of the CMV.SAR vector in transduced cells

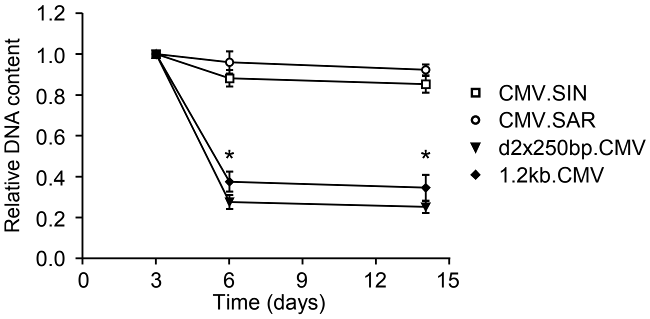

293T cells were transduced at MOI 1 (relative DNA titer) with four vectors utilizing the CMV-promoter. After 3, 6, 14 and 27 days cells were harvested and the proviral load determined by qPCR using primers LV2 and ALB. The vectors containing two separated copies of the insulator (d2x250bp.CMV and 1.2kb.CMV) elicit a significant (*, $p<0.05$) drop in proviral load within the first 6 days after transduction, compared to the control vector, CMV.SIN. In contrast, the proviral load of the CMV.SAR vector does not drop during this time period. The experiment was continued for 27 days with no change in proviral load compared to the 14 days time point. Error bars denote standard error of the mean.
